# Single-cell RNA sequencing reveals SARS-CoV-2 infection dynamics in lungs of African green monkeys

**DOI:** 10.1126/scitranslmed.abe8146

**Published:** 2021-01-11

**Authors:** Emily Speranza, Brandi N. Williamson, Friederike Feldmann, Gail L. Sturdevant, Lizzette Pérez- Pérez, Kimberly Meade-White, Brian J. Smith, Jamie Lovaglio, Craig Martens, Vincent J. Munster, Atsushi Okumura, Carl Shaia, Heinz Feldmann, Sonja M. Best, Emmie de Wit

**Affiliations:** 1 Laboratory of Virology, Division of Intramural Research, National Institute of Allergy and Infectious Diseases, National Institutes of Health, Hamilton, MT 59840, USA.; 2 Laboratory of Immune System Biology, Lymphocyte Biology Section, Division of Intramural Research, National Institute of Allergy and Infectious Diseases, National Institutes of Health, Bethesda, MD 20814, USA.; 3 Rocky Mountain Veterinary Branch, Division of Intramural Research, National Institute of Allergy and Infectious Diseases, National Institutes of Health, Hamilton, MT 59840, USA.; 4 Research Technologies Branch, Division of Intramural Research, National Institute of Allergy and Infectious Diseases, National Institutes of Health, Hamilton, MT 59840, USA.

## Abstract

Detailed knowledge about the dynamics of severe acute respiratory syndrome coronavirus 2 (SARS-CoV-2) infection is important for uncovering the viral and host factors that contribute to coronavirus disease 2019 (COVID-19) pathogenesis. Old-World nonhuman primates recapitulate mild to moderate cases of COVID-19, thereby serving as important pathogenesis models. We compared African green monkeys inoculated with infectious SARS-CoV-2 or irradiated, inactivated virus to study the dynamics of virus replication throughout the respiratory tract. Genomic RNA from the animals inoculated with the irradiated virus was found to be highly stable, whereas subgenomic RNA, an indicator of viral replication, was found to degrade quickly. We combined this information with single-cell RNA sequencing of cells isolated from the lung and lung-draining mediastinal lymph nodes and developed new analysis methods for unbiased targeting of important cells in the host response to SARS-CoV-2 infection. Through detection of reads to the viral genome, we were able to determine that replication of the virus in the lungs appeared to occur mainly in pneumocytes, whereas macrophages drove the inflammatory response. Monocyte-derived macrophages recruited to the lungs, rather than tissue-resident alveolar macrophages, were most likely to be responsible for phagocytosis of infected cells and cellular debris early in infection, with their roles switching during clearance of infection. Together, our dataset provides a detailed view of the dynamics of virus replication and host responses over the course of mild COVID-19 and serves as a valuable resource to identify therapeutic targets.

## A dynamic response to SARS-CoV-2

While a number of animal models for SARS-CoV-2 have been developed, nonhuman primates are among the best at recapitulating human COVID-19. Here, Speranza *et al*. used single-cell RNA sequencing to demonstrate that SARS-CoV-2 replicates in the lung of African green monkeys and that immune cell populations in the lung change over the course of infection. Sequencing data revealed that pneumocytes are the site of viral replication in the lung, and infiltrating macrophages are responsible for clearing infected cells and cellular debris early in infection. Together, these findings further our understanding of the dynamics of SARS-CoV-2 infection and host immune responses in vivo.

## Teaser

Single-cell RNA sequencing of lungs of SARS-CoV-2–infected African green monkeys reveals virus replication and host response dynamics in vivo.

## INTRODUCTION

A wealth of clinical and laboratory studies have been reported concerning severe acute respiratory syndrome coronavirus 2 (SARS-CoV-2), the causative agent of coronavirus disease 2019 (COVID-19) (*1*, *2*). A key issue among the many unanswered questions that remain involves the dynamics of SARS-CoV-2 infection, including the identity of the cells that support active virus replication and the immune cells that contribute to the response to infection. Although multiple cell types in the respiratory tract have been reported to express the critical receptor needed for entry, angiotensin-converting enzyme 2 (ACE2), as well as the transmembrane protease, serine 2 (TMPRSS2) needed to initiate replication (*3*, *4*), it is not clear which of these cell types is the site of SARS-CoV-2 replication. One study using a new algorithm (Viral Track) to detect viral reads in single-cell sequencing samples suggested that both epithelial cells and macrophages contain viral RNA in human bronchoalveolar lavage fluid (BALF) (*5*). However, because the presence of genomic RNA (gRNA) alone does not equate to productive virus infection, this study did not address whether this detection of RNA was the result of virus replication in these cells.

Beyond viral replication dynamics, a thorough study of the host response to infection at the major site of virus replication, the lungs, is needed to better understand potential causes of organ damage and targets for therapeutic intervention. The use of single-cell technologies such as multiparameter flow cytometry and single-cell sequencing allows for analysis of individual cell states. Sequencing of single cells in BALF (*6*) and upper respiratory tract swabs (*7*) collected from patients with COVID-19 has detected markers of severe disease in hospitalized individuals. Others have examined the immune response to infection by using single-cell sequencing to profile peripheral blood mononuclear cell samples from patients with COVID-19 (*8*, *9*). However, both approaches have limited power to address the dynamics of infection, the host response in the lungs, the main site of virus replication, and disease pathogenesis. Furthermore, neither strategy provides insight into the immune response in the lung-draining lymph nodes.

The limitations of human studies with respect to time of sampling relative to exposure, differences in exposure dose and route, and capacity for analyzing tissues in-depth can be overcome using animal models, albeit currently at the cost of not fully replicating the severe disease observed in humans. Multiple animal models of SARS-CoV-2 infection are being developed to test therapeutics and vaccines and to better understand the dynamics of COVID-19 disease progression and the immune response to infection in a time-resolved manner. Nonhuman primates are often used as models for infection and pathogenesis because they often recapitulate human disease. African green monkeys are a commonly used nonhuman primate model for studies of respiratory viruses, including SARS-CoV (*10*). Recently, two studies showed that inoculation of African green monkeys with SARS-CoV-2 results in mild respiratory disease with virus detected in the upper and lower respiratory tract, suggesting that African green monkeys are a suitable nonhuman primate disease model to study SARS-CoV-2 infection and host response dynamics (*11*, *12*).

Here, we used traditional virological methods, single-cell RNA sequencing (scRNA-seq), and immunohistopathology to address some of the major questions about SARS-CoV-2 replication and host responses. Our combined approach, using inoculation with infectious and inactivated SARS-CoV-2, determined which cell types are productively infected and assessed the host response to virus replication in the lung. Together, the result is an emerging picture of the viral and immune events associated with mild COVID-19 disease, aiding our understanding of the dynamics of SARS-CoV-2 infection with high resolution.

## RESULTS

### sgRNA, but not gRNA, detection reveals active virus replication in the respiratory tract of SARS-CoV-2–infected African green monkeys

**Fig. 1:**
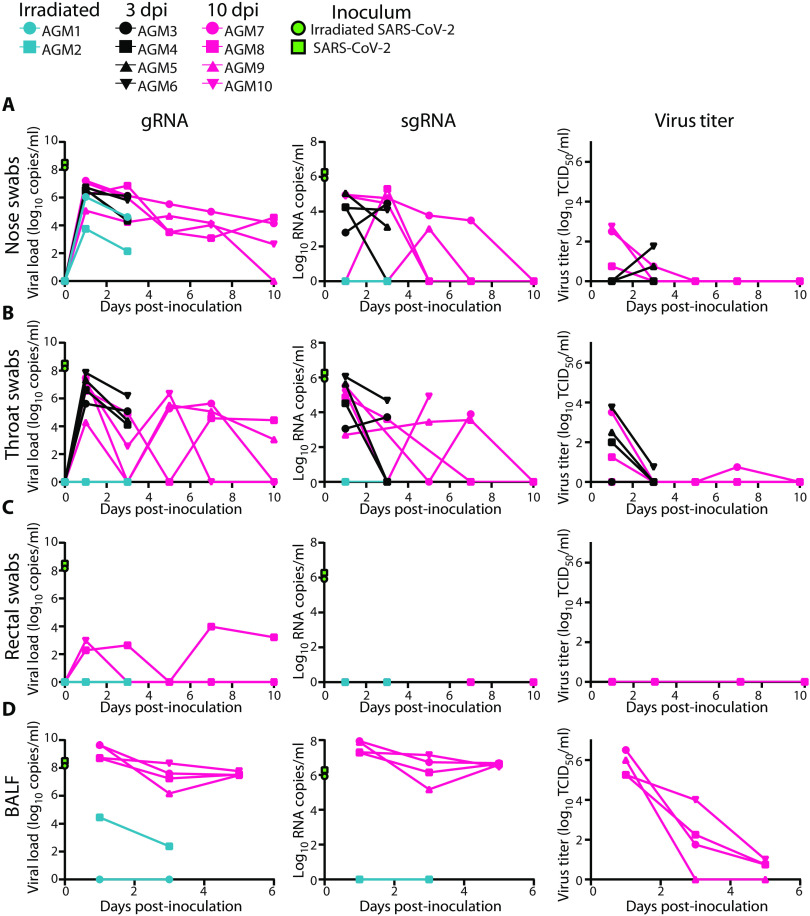
Viral loads and virus titers in swabs and BALF from African green monkeys. Two African green monkeys were inoculated with γ-irradiated SARS-CoV-2 (*n* = 2). Eight African green monkeys were inoculated with infectious SARS-CoV-2 isolate nCoV-WA1-2020. After inoculation, clinical exams were performed during which nose (**A**), throat (**B**), and rectal swabs (**C**) were collected; (**D**) bronchoalveolar lavages were performed at 1, 3, and 5 dpi on the four animals remaining in the study through 10 dpi; and viral loads and titers were measured. qRT-PCR was performed to detect gRNA (left column) and sgRNA (middle column), and in vitro virus titration was performed to detect infectious virus (right column) in these samples. Amount of gRNA and sgRNA in the inocula (γ-irradiated and infectious) is indicated at time point zero. Teal: animals inoculated with γ-irradiated virus; black: animals inoculated with infectious virus and euthanized at 3 dpi; pink: animals inoculated with infectious virus and euthanized at 10 dpi.

To study SARS-CoV-2 infection and its consequences, two groups of four African green monkeys were inoculated with a total dose of 2.6 × 10^6^ 50% tissue culture infectious dose (TCID_50_) of replication-competent virus, whereas two control animals were inoculated with virus inactivated by γ-irradiation. Clinical signs in the control animals were limited to reduced appetite, likely as a response to repeated anesthesia and intubation, on 0 and 1 days post-inoculation (dpi) (Table 1). Tachypnea was observed in five of eight animals inoculated with infectious SARS-CoV-2. In these animals, disease was mild to moderate and transient, with animals recovering between 5 and 9 dpi (fig. S1A and Table 1). At 1 and 3 dpi for all animals and 5, 7, and 10 dpi for four of the animals, we collected nose, throat, and rectal swabs. High amounts of viral gRNA were detected in nose and throat swabs after inoculation with SARS-CoV-2 and declined over time (Fig. 1, A and B). Rectal swabs were positive for gRNA at most time points for one animal with a severely reduced appetite (AGM8) (Fig. 1C).

**Table 1:** Clinical signs observed in African green monkeys inoculated with irradiated or infectious SARS-CoV-2. Animals were observed daily according to a standardized scoring sheet (*19*); the same person assessed the animals throughout the study. N/A, not available.

**Inoculum**	**Animal**	**Clinical signs observed** **1 to 3 dpi**	**Clinical signs observed** **4 to 10 dpi**	**Observations at necropsy**
Irradiated SARS-CoV-2	AGM1	Reduced appetite.	N/A	None.
		Euthanized 3 dpi.		
	AGM2	Reduced appetite.	N/A	None.
		Euthanized 3 dpi.		
SARS-CoV-2	AGM3	Reduced appetite.	N/A	Gross lung lesions; cervical and mediastinal lymph nodes enlarged.
		Euthanized 3 dpi.		
	AGM4	Tachypnea; reduced appetite.	N/A	Gross lung lesions; mediastinal lymph nodes mildly enlarged.
		Euthanized 3 dpi.		
	AGM5	Tachypnea; reduced appetite.	N/A	Gross lung lesions; mediastinal lymph nodes enlarged.
		Euthanized 3 dpi.		
	AGM6	Reduced appetite.	N/A	Gross lung lesions; mediastinal lymph nodes enlarged.
		Euthanized 3 dpi.		
	AGM7	Reduced appetite.	Reduced appetite.	Gross lung lesions; mediastinal lymph nodes enlarged.
			Recovered at 5 dpi.	
	AGM8	Hunched posture; tachypnea; severely reduced appetite.	Hunched posture; tachypnea; severely reduced appetite.	Lungs consolidated; mediastinal lymph nodes enlarged and hemorrhagic.
			Recovered at 9 dpi.	
	AGM9	Tachypnea; reduced appetite.	Tachypnea; reduced appetite.	Gross lung lesions; mediastinal lymph nodes enlarged.
			Recovered at 6 dpi.	
	AGM10	Hunched posture; tachypnea; coughing; reduced appetite.	Reduced appetite.	None.
			Recovered at 5 dpi.	

Nasal swabs, although not throat or rectal swabs, from control animals inoculated with γ-irradiated virus contained high amounts of gRNA at 1 dpi and were still positive at 3 dpi. To determine whether detection of subgenomic RNA (sgRNA) would be able to distinguish between RNA originating from the inoculum from that derived from replicating virus, all swabs positive for gRNA were evaluated by quantitative real-time reverse transcription polymerase chain reaction (qRT-PCR) to detect sgRNA. Although sgRNA derived from infected, lysing cells in the cell culture in which the virus stock was produced was present at high copy numbers in both inocula, sgRNA could not be detected in swabs collected from control animals. sgRNA was detected in nose and throat swabs from animals inoculated with infectious virus (Fig. 1, A and B), indicating that sgRNA likely reflects that virus replication occurred and that gRNA is highly stable, especially in the nasal cavity. Infectious virus could be detected by virus titration early after inoculation in nose and throat swabs; no infectious virus could be detected in rectal swabs (Fig. 1C). As a measure of virus replication in the lower respiratory tract, we collected BALF from the two control animals at 1 and 3 dpi and at 1, 3, and 5 dpi from the four SARS-CoV-2–infected animals euthanized at 10 dpi. gRNA could be detected on 1 and 3 dpi in one of the two control animals; however, sgRNA could not be detected. High copy numbers of gRNA and sgRNA were detected in BALF from the four infected animals evaluated, in line with detection of infectious virus through 5 dpi (Fig. 1D).

### Virus replication is mostly confined to the lowery respiratory tract in African green monkeys

At 3 dpi, the two control animals and four of the SARS-CoV-2–infected animals were euthanized. The remaining four SARS-CoV-2 animals were euthanized at 10 dpi. Upon necropsy, lungs were examined for gross lesions. No abnormalities were detected in the lungs of the two control animals. At 3 dpi, all four animals inoculated with active SARS-CoV-2 showed varying degrees of gross lung lesions and enlarged mediastinal lymph nodes (Table 1 and fig. S1B). By 10 dpi, one animal did not show gross abnormalities, whereas the other three animals showed gross lung lesions and enlarged mediastinal lymph nodes (Table 1 and fig. S1B). Tissue samples from these animals were assessed for the presence of gRNA and sgRNA. Viral gRNA loads were highest in samples collected from the lung lobes and were higher at 3 dpi than 10 dpi. Despite high copy numbers of sgRNA in lung tissue through 10 dpi, virus could only be isolated at 3 dpi (fig. S1C and table S1), indicating that in tissue, sgRNA is a much more sensitive detection method than virus isolation in tissue culture. Analysis of other respiratory tract tissues showed that although gRNA can be detected in all tested sites early after inoculation, sgRNA can be detected consistently only in the trachea and right bronchus (fig. S1D).

We also analyzed tissues of the gastrointestinal (GI) tract for the presence of viral RNA. Only gRNA could be detected in the GI tract of several animals after inoculation with SARS-CoV-2 at 3 and 10 dpi. However, in AGM8, the animal with severely reduced appetite, high copy numbers of both gRNA and sgRNA could be detected in duodenum, jejunum, ileum, cecum, and colon (fig. S1E) and virus was isolated from the ileum and cecum (table S1). Histologically, the intestinal tract from this animal appeared normal. However, immunohistochemistry (IHC) imaging revealed epithelial cells containing SARS-CoV-2 antigen in the ileum of AGM8 (fig. S2, A to C).

**Fig. 2:**
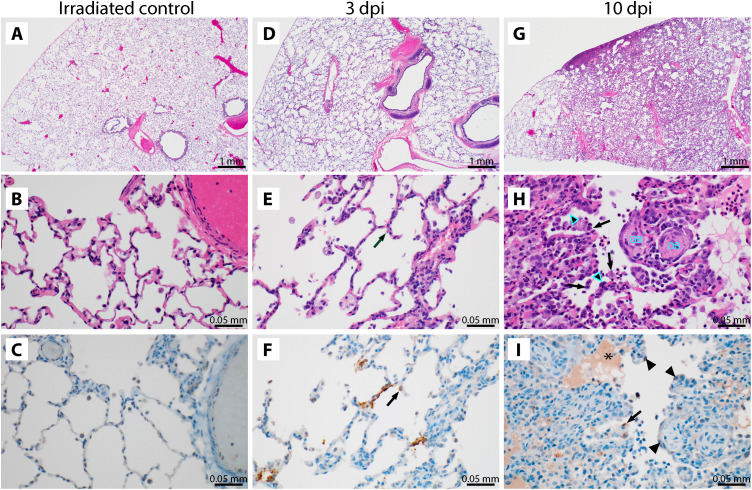
Histological changes are observed in the lungs of African green monkeys inoculated with SARS-CoV-2. (**A** to **C**) African green monkeys were inoculated with γ-irradiated SARS-CoV-2 (*n* = 2) and euthanized at 3 dpi; eight animals were inoculated with SARS-CoV-2 isolate nCoV-WA1-2020. (**D** to **F**) Four of those were euthanized at 3 dpi. (**G** to **I**) The remaining four animals were euthanized at 10 dpi. Histological analysis was performed on lung tissue from all animals. (A) Lungs of animals inoculated with γ-irradiated SARS-CoV-2 were normal at 3 dpi. (B) This was further confirmed at high magnification. (C) No SARS-CoV-2 antigen could be detected in lungs from animals inoculated with γ-irradiated SARS-CoV-2. (D) Mildly thickened septa were observed at 3 dpi in animals inoculated with infectious SARS-CoV-2. (E) Alveolar septa are slightly thickened and more cellular at 3 dpi. (F) Cytoplasmic and membrane-associated viral antigen in pneumocytes at 3 dpi. (G) Discrete foci of interstitial pneumonia are apparent at the periphery of the lung at 10 dpi. (H) Alveolar edema (*), type II pneumocyte hyperplasia (arrowheads), increased alveolar macrophages (arrows), and infiltrating lymphocytes and neutrophils are observed at 10 dpi, as well as proliferative nodules associated with terminal airways resembling obstructive bronchiolitis (OB). (I) Rare viral antigen could be detected in mononuclear cells, presumably alveolar macrophages, with cytoplasmic debris (arrows) at 10 dpi; background blush is observed in alveolar proteinaceous fluid (*), but pneumocytes do not exhibit immunoreactivity (arrowheads). Magnification, ×20 (scale bars: 1 mm) (A to C) and ×400 (scale bars: 0.05 mm) (D to I).

Histological analysis of the lungs of the two control animals showed no abnormalities (Fig. 2, A to C). The lungs of the four animals inoculated with SARS-CoV-2 and euthanized at 3 dpi showed subtle alveolar thickening, indicative of an early inflammatory response (Fig. 2, D to F). Viral antigen could be detected by IHC in type I pneumocytes and alveolar macrophages of all four animals. Alveolar thickening was still visible in the four animals inoculated with SARS-CoV-2 and euthanized at 10 dpi. Two of these animals showed histopathological changes consistent with interstitial pneumonia frequently centered on terminal bronchioles and early lesions in terminal airways, resembling obstructive bronchiolitis (Fig. 2, G to I). At this time, viral antigen could only be detected in type I pneumocytes and alveolar macrophages of one of four animals (AGM10). Three of four mediastinal lymph nodes from the 10 dpi samples exhibited a mild to moderate follicular hyperplasia, and the lymph nodes of all four animals exhibited rare mononuclear cell immunoreactivity (fig. S2, D and E).

### RNA sequencing of single cells identified pneumocytes as the main site of productive virus replication

**Fig. 3:**
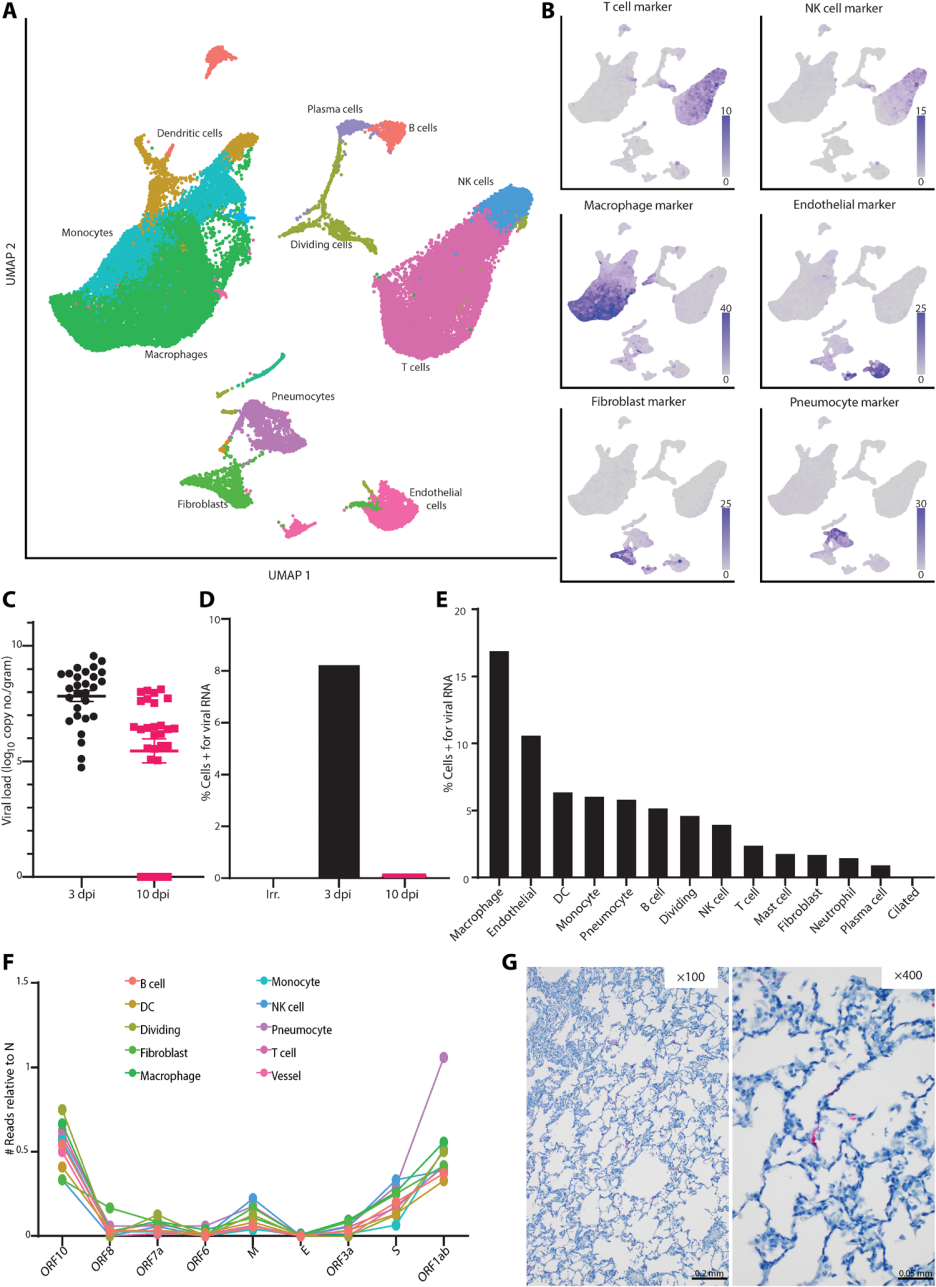
Single-cell sequencing reveals viral dynamics in lung tissue. (**A**) UMAP projection of scRNA-seq data from whole lung sections from all 10 animals combined. Each point is an individual cell; colors are based on cell type annotation. Cell names are shown next to their largest cluster. NK, natural killer. (**B**) Validation of cell type identities using marker gene sets. The intensity of the purple color represents higher expression of the indicated marker set. Gray coloring indicates that the cell did not express any genes in the marker set. (**C**) Viral load in cells isolated from the lungs was evaluated via qRT-PCR for gRNA and grouped for all lobes across each animal in the indicated groups. (**D**) The percentage of cells identified by scRNA-seq that were positive for any reads aligning to the viral genome by dpi is reported. (**E**) Percentage of cells from the 3-dpi samples positive for any reads aligning to the viral genome grouped by cell type. DC, dendritic cell. (**F**) The number of cells grouped by cell type with reads aligning to other locations across the viral genome, all normalized to the number of cells expressing nucleocapsid (N) gene. M, membrane; E, envelope; S, spike. Genes are ordered from the 3′ to 5′ end of the SARS-CoV-2 genome. (**G**) ISH for viral spike RNA in lung tissues at 3 dpi. Viral RNA staining is shown in red at ×100 magnification (scale bar: 0.2 mm) and ×400 magnification (scale bar: 0.05 mm).

On the day of necropsy, we collected sections of the lungs of each animal that contained an active lesion, except in animals where gross lung lesions were not observed at necropsy (Table 1). These sections were processed directly after necropsy into single-cell suspensions, and scRNA-seq was conducted using complementary DNA (cDNA) generated immediately without freezing or fixation of the cells, thereby allowing collection of whole-cell data. This allowed for high-quality single-cell data to be collected with a high fraction of reads in cells (>80%) and a low fraction of cells enriched in mitochondrial genes (<5%). Uniform manifold approximation and projection (UMAP) was used to display the data, with each cell annotated with its likely cell identity (Fig. 3A). For the latter analysis, we developed an algorithm to perform unbiased cell classification, which is a correlation-based method that uses transcriptional profiles from annotated lung tissue and determines the most likely identity for an individual cell or a cluster of cells and learns from the local neighbor information for each cell its most likely identity. To validate the results, we built a marker gene set for each cell type of interest, showing that the expected gene expression profile matched the cell type annotation (Fig. 3B). Using these assignments, we compared the percentage of cells in each sample belonging to each annotation and found an increase in the number of pneumocytes (*P* value of 10 dpi to irradiated = 0.06) and dividing cells (*P* value of 10 dpi to irradiated = 0.04) as infection progressed (fig. S3). This is consistent with the histology results showing an influx of inflammatory cells and type II pneumocyte hyperplasia at 10 dpi (Fig. 2F).

Because SARS-CoV-2 is a polyadenylated virus, with such modification present in both the genome (gRNA) and the transcripts (sgRNA), we could map reads to both the African green monkey and the SARS-CoV-2 genomes and determine which cell types contained viral RNA. The scRNA-seq data showed a similar pattern to the qRT-PCR data described, with the highest percentage of positive cells identified at 3 dpi and decreasing by 10 dpi (fig. S1C). Furthermore, no viral RNA was detected in the animals inoculated with γ-irradiated virus (Fig. 3, C and D). We then parsed these data down to individual cell types. At 3 dpi, viral RNA could be detected in several cell types, with the macrophage population having the highest percentage of cells positive for viral RNA (Fig. 3E). The viral RNA in these cells could be due to virus replication, phagocytosis of infected cells, abortive infections, or having virus particles partitioned with cells during creation of gel beads in emulsion in the 10X Genomics processing steps. To discriminate between cells supporting active virus replication versus those containing viral RNA due to other processes, we looked at the distribution of reads across the genome. Because of the 3′ bias of sequencing with the 10X platform, we saw an expected enrichment of reads in the 3′ end (fig. S4A), with a bias to the location of the nucleocapsid (N) gene in the genome. In addition, in the pseudo-bulk data read pileups, we were able to detect small enrichments of reads in specific areas at the most 3′ end of all the transcripts, including open reading frame (ORF) 1ab, as well as pileups in the first 5000 base pairs (bp) of the ORF1ab region (fig. S4B). These points of enrichment in the first 5000 bp matched to locations of noncanonical sgRNA formed by a jump of the RNA-dependent RNA polymerase in ORF1ab to N in the SARS-CoV-2 transcriptome and were suggestive of actively replicating virus (*13*). On the basis of these findings, we examined each cell type for reads showing such evidence of subgenomic transcripts involving a region other than the N gene. We observed an expected ratio of gene counts across the SARS-CoV-2 genome, where genes closer to N and longer genes had higher counts associated with them across all cell types. Only one annotated cell type, pneumocytes, had an abnormally high proportion of cells that were positive for the N gene and for ORF1ab when compared to all other cell types (Fig. 3F). This suggests that despite many cell types containing viral RNA, pneumocytes were likely the dominant cell type supporting productive viral replication, as higher than expected counts to ORF1ab are suggestive of high amounts of sgRNA and noncanonical sgRNA. To examine this hypothesis further, we performed IHC and in situ hybridization (ISH) on lung tissues at 3 dpi. Although a few macrophages were positive for viral antigen by IHC (Fig. 2F), only pneumocytes were positive by ISH for the viral genome, consistent with the notion that this is the only cell type analyzed supporting active virus replication in the lungs of African green monkeys (Fig. 3G).

### Infection-related changes occur in the transcriptional states of cells isolated from the lungs of African green monkeys

To gain insight into the biological effects of SARS-CoV-2 infection on diverse cells in the lungs, we examined the transcriptional signatures in cells recovered from animals under each of the viral exposure conditions. To this end, we developed a new algorithm in which each population of cells was examined for a clustering bias in a pairwise comparison along individual principal components (PCs). We then used the PC information and gene set enrichment analysis to determine gene signatures driving the differences between two experimental groups. Using this method, we found that macrophages showed large clustering biases in all the comparisons, suggesting that they had the biggest transcriptional shift over the course of infection (fig. S5). The 10-dpi sample changes also showed changes in plasma cells and pneumocytes relative to 3 dpi, likely due to changes in cell numbers as described above (fig. S5).

**Fig. 4:**
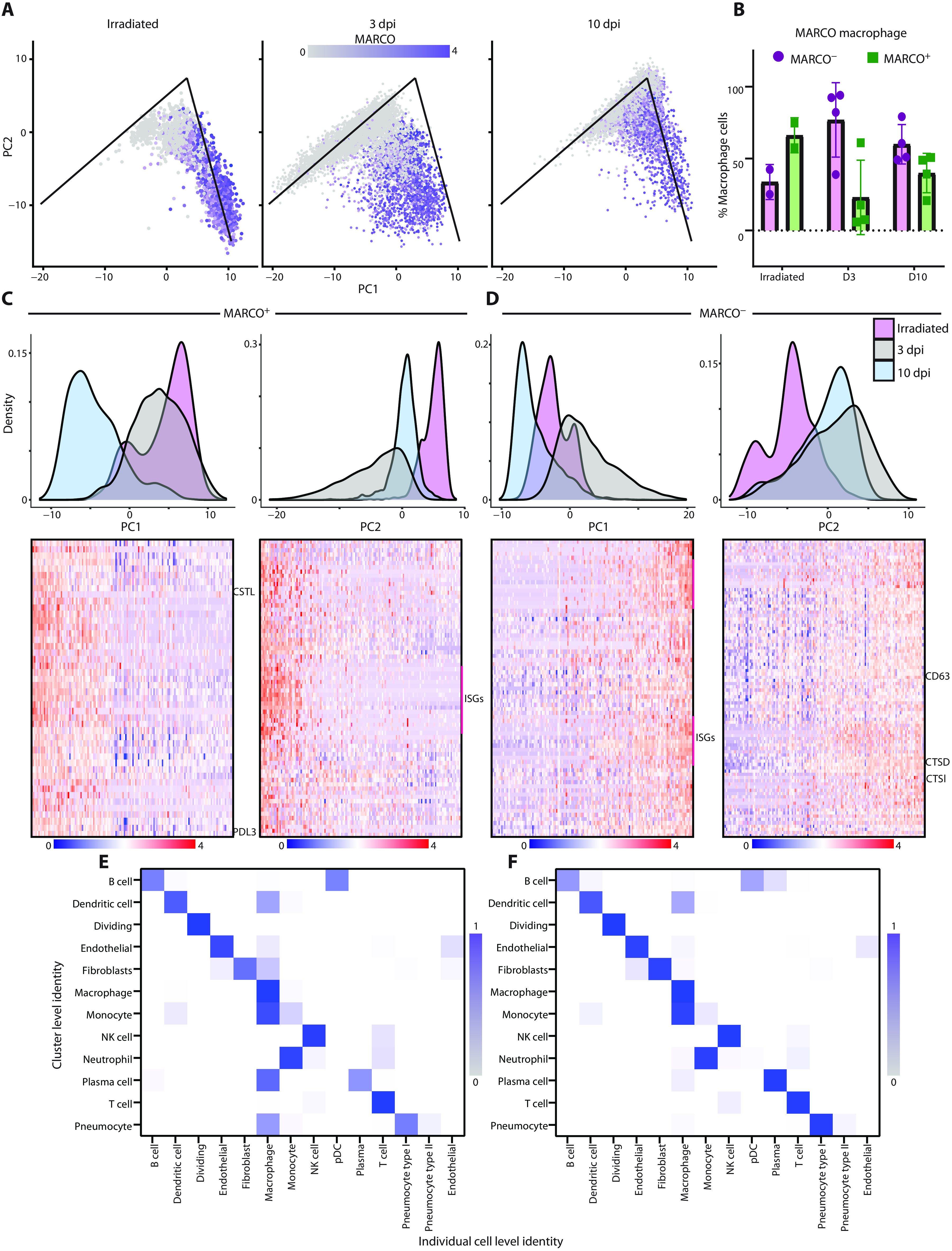
Macrophage populations in the lungs are dynamic during SARS-CoV-2 infection. (**A**) Graphs depicting PC analysis of lung macrophages. The *x* axis is PC1, and the *y* axis is PC2. Experimental groups are plotted independently. Each point is an individual cell and is colored on the basis of the expression of MARCO. The lines on the PC graphs are for reference across the samples and represent matching locations. (**B**) Quantification of the percentage of macrophages that are MARCO^−^ (purple) or MARCO^+^ (green) across the three different experimental groups. (**C**) The MARCO^+^ macrophage PC analysis (density plots) was plotted by histograms representing the experimental groups. PC1 is shown on the left, and PC2 is shown on the right. The heatmap below showing the individual cells (columns) sorted based on their location along PC1 or PC2. Top genes showing high correlation along that PC are clustered in rows. A few of the gene names are noted just to the right of the heatmaps. (**D**) The density plots and histograms are shown as in (C) for MARCO^−^ cells. (**E** and **F**) Comparison between the individual cell identity (columns) and the cluster identity (rows) based on an unbiased identification algorithm at 3 dpi (E) or 10 dpi (F) for SARS-CoV-2−infected animals. The color intensity represents the percent of individual cells in the cluster that match the identified phenotype.

The macrophage analysis was extended by selecting and reanalyzing cells that had either a macrophage- or monocyte-like phenotype and further classified to cells with a more tissue-resident phenotype, indicative of alveolar macrophages, or a more monocyte-derived phenotype, indicative of interstitial macrophages and monocytes using the expression of the macrophage receptor with collagenous structure (MARCO) gene (Fig. 4A). The MARCO gene was selected as it is highly expressed in alveolar macrophages and is absent in interstitial macrophages or monocytes based on previous data in human lung samples (*14*). A comparison of the percentage of the MARCO^+^ to MARCO^−^ cells showed that, at 3 dpi, there was a large influx of monocyte-derived cells, as most of the macrophages detected at that time were MARCO^−^, consistent with pathology findings in human samples (*15*). This shift began to normalize by 10 dpi as the animals recovered (Fig. 4B).

To identify the major pathways whose genes were responsible for the analytical differences between these two cell populations, we performed a similar subcluster analysis on the MARCO^+^ versus MARCO^−^ cells (table S2). For the MARCO^+^ cells, there was a transcriptional shift at 10 dpi along PC1 (Fig. 4C). Using gene set enrichment analysis, we found pathways associated with lysosomes and extracellular matrix modification enriched along this component, which are also enriched during active phagocytosis (fig. S6A). This suggests that these macrophages were active in clearing cell debris from the infected lungs, potentially explaining why the macrophages stain for viral antigen at this time point (Fig. 2I). Comparatively, along PC2, we see a shift at 3 dpi and, to a lesser extent, at 10 dpi. The genes associated with this component were enriched for pro-inflammatory pathways, including the gene set of interferon-stimulated genes (Fig. 4C and fig. S6B). In distinction to the MARCO^+^ cells, we saw a shift for MARCO^−^ cells along PC1, mostly associated with pro-inflammatory pathways in samples at 3 dpi (Fig. 4D and fig. S6C). Along PC2 in the MARCO^−^ cells, we see similar pathways that were found in the MARCO^+^ cells along PC1, suggesting an active phagocytosis state, as well as migratory genes (CD63) (fig. S6, D and E). This was observed at both 3 and 10 dpi (Fig. 4D). These data suggest that the macrophages were in an inflammatory state at 3 dpi that was beginning to resolve by 10 dpi and that MARCO^−^ macrophages, but not MARCO^+^ macrophages, were primarily involved in cleanup of cells and cellular debris at 3 dpi.

To assess interactions between macrophages and other cell types, we looked at a combination of the cluster identity as compared to the individual cell identity. Using the same cell identity algorithm and the clusters calculated by Seurat (*16*), we looked within individual clusters to identify which cells had a strong macrophage gene marker. At 3 dpi, the clusters associated with pneumocytes, fibroblasts, and endothelial cells all had strong gene markers for macrophages, suggesting that these cells were being actively phagocytosed by the macrophages (Fig. 4E). This signature was mostly absent by 10 dpi, possibly because the MARCO^+^ macrophages were picking up more dead cells and cellular debris by this time rather than whole cells with replicating virus (Fig. 4F).

### Mediastinal lymph nodes are in an inflammatory state at 3 dpi

**Fig. 5:**
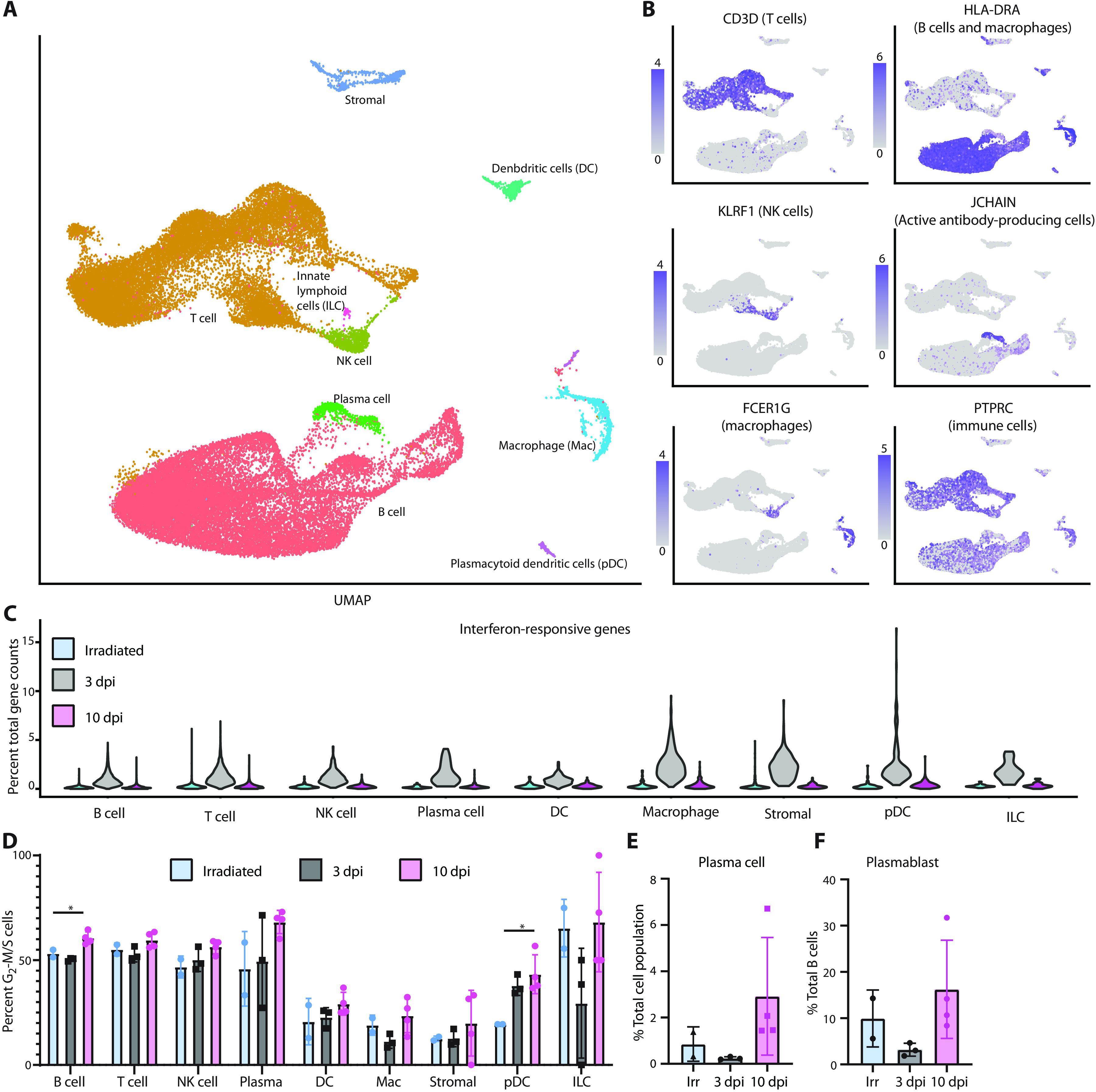
Single-cell sequencing of mediastinal lymph nodes shows resolution of inflammatory response. (**A**) UMAP projection of single-cell sequencing data from cells isolated from the mediastinal lymph nodes of all 10 animals combined. Each point represents an individual cell, and cells are colored on the basis of their cell type. The names of the cell types are placed next to their largest cluster. (**B**) Single gene expression analysis was used to validate cell type identifications. (**C**) Percent of total gene counts for each cell for a subset of interferon-responsive genes (*y* axis). The *x* axis denotes cell types and experimental group. (**D**) Percentage of each cell population (*x* axis) that is actively dividing (stage G_2_-M or S) as determined by a profile of gene expression. Each point is an individual animal, and bars represent the mean and SD of the samples. (**E**) The percentage of plasma cells in each sample compared relative to the total cell number is plotted. (**F**) The percentage of plasmablast cells relative to the number of B cells is plotted. *P < 0.05, one-way ANOVA.

To relate these virus replication and inflammatory changes in the lungs to changes in secondary lymphoid tissues, we sampled cells from the mediastinal lymph nodes. We collected whole mediastinal lymph nodes from animals at the time of necropsy and prepared the samples along with the lung tissue. We were not able to detect any viral RNA in the lymph node by scRNA-seq, despite a few mononuclear cells staining for viral antigen by IHC (fig. S2E). This could be due to low abundance of RNA, low sensitivity of the scRNA-seq assay in its ability to detect lowly abundant transcripts, or an inability to capture high abundance of nonlymphocyte populations from the lymph node when generating cell suspensions (*17*). Using an annotation strategy like that used for lung cells, we could detect most of the major cell populations of the lymph node, with most cells annotating as T or B lymphocytes (Fig. 5A). Again, as with the lung, the annotated cell types expressed known marker genes associated with their phenotype (Fig. 5B). The largest transcriptional change observed across all the cell types was an increase in interferon-responsive genes at 3 dpi, suggesting an inflammatory state in the draining lymph nodes. This transcriptional profile was resolved by 10 dpi and was absent in the lymph nodes from animals that received γ-irradiated virus (Fig. 5C). The genes characterized in the set at 3 dpi include many classical type I interferon–responsive genes (table S2) that are up-regulated across many viral infections.

There was an increase in the percentage of dividing cells (cells identified as in G_2_-M or S phase) among B cells (*P* value of 10 dpi to irradiated = 0.02) and plasmacytoid dendritic cells (*P* value of 10 dpi to irradiated = 0.02) as infection progressed (Fig. 5D). This is consistent with histology findings of lymph node hyperplasia later at 10 dpi (fig. S2D). Evaluation of antibody-secreting cells revealed an increase in plasma cells (*P* value of 10 to 3 dpi = 0.03; Fig. 5E). The development of plasmablasts at 3 dpi in animals that received SARS-CoV-2 was lacking (Fig. 5F), potentially due to the inflammatory state of the lymph node and early time of collection. When looking at which cell type showed the strongest transcriptional changes, we found that even at 10 dpi, macrophages showed the strongest transcriptional shift when compared to samples from animals inoculated with γ-irradiated virus (fig. S7A). Within the macrophage population, we saw an up-regulation in a small subset of macrophages with markers for fully mature monocyte-derived macrophages, such as Chitinase 1, at 10 dpi (fig. S7B).

Further analysis of the T cells in the mediastinal lymph nodes and their relation to the lung T cell compartment was performed. To identify CD4 versus CD8 T cells, we used a similar matching algorithm as above, using splenic T cells as background (*14*). In both the lung and lymph node T cell compartment, we observed an increase in the percentage of CD4^+^ T cells in the lung (*P* = 0.003) and lymph node (*P* = 0.0026) at 10 dpi relative to irradiated control lungs (fig. S8, A and B). We also saw a decrease in proportion of CD8^+^ T cells at 10 dpi relative to irradiated control lungs (*P* = 0.0009) and lymph nodes (*P* = 0.008; fig. S8, A and B). No change in the regulatory T cell compartment was observed (fig. S8, A and B). We were able to identify a small subset of cells enriched for genes associated with T follicular helper genes that showed an expansion at 10 dpi in the lymph node (fig. S8C). These cells are often associated with anti-inflammatory cytokines (such as interleukin-10), which again further suggests that the inflammatory state observed in the lymph node and lung at 3 dpi is being cleared by 10 dpi.

## DISCUSSION

This study used large-scale single-cell sequencing in a nonhuman primate model of SARS-CoV-2 infection. The benefit of using animal models to study SARS-CoV-2 lies in the ability to collect time-resolved datasets of the lungs instead of being limited to sampling at terminal time points (*18*, *19*). Using traditional virological methods and scRNA-seq, we have begun to parse out the infection dynamics that occur as the disease progresses and virus is eventually cleared in the African green monkey model of mild COVID-19. These findings are consistent with other studies done in African green monkeys (*11*, *12*), which share a similar disease pathology to that seen with rhesus macaques (*20*) and cynomolgus macaques (*21*). In all cases, the animals display mild disease signs leading to clearance of the virus and recovery.

One unique aspect of this study was the inclusion of two animals inoculated with γ-irradiated SARS-CoV-2, which renders the virus unable to replicate (*22*). Unlike previous studies in nonhuman primate models of disease, this enabled us to directly compare the detection of viral gRNA from replicating versus nonreplicating virus. We found that gRNA was highly stable even in the absence of replicating virus and was detectable at 1 and 3 dpi in nose swabs and BALF from the animals inoculated with γ-irradiated virus, whereas the sgRNA appeared to degrade quickly in swabs in the absence of virus replication. Such viral dynamics could be due to gRNA being encapsulated or due to secondary structures that help prevent degradation (*23*). This has potential implications in patient testing, as positive gRNA results by PCR may not represent replication-competent SARS-CoV-2, especially because the highest amount of gRNA detected in the samples from the animals inoculated with irradiated virus were found in the nasal swabs currently used for most patient diagnostics. Our data suggest that PCR-based tests, which specifically target SARS-CoV-2 sgRNA, may provide a more realistic signature of replicating virus than detection of gRNA, improving models to determine patient infectivity (*24*, *25*). Because animals cleared sgRNA faster than gRNA, the duration of time PCR-positive patients needs to remain in isolation could potentially be reduced.

Using the African green monkey model, we have been able to explore the dynamics of SARS-CoV-2 infection at the time of peak disease in the lungs when most virus replication is occurring. Because of the nature of the 10X Genomics platform and the polyadenylation of the SARS-CoV-2 genome and sgRNA, we were able to determine which cell types were positive for viral RNA in a method similar to previously published reports (*5*), although we did not include any other alignments beyond SARS-CoV-2. To expand upon detection of viral RNA reads, we looked at the distribution of the reads across the genome to determine whether we could find a unique pattern of alignment associated with replicating virus. Further investigation into the distribution of reads across the genome suggests that productive virus replication is mostly occurring in infected pneumocytes, although the macrophage population contained the highest percentage of cells positive for viral RNA. These data provide evidence that macrophages do not support efficient virus replication in vivo. A potential reason that the macrophages could have such high viral RNA and immunoreactivity without supporting full replication is through phagocytosis of virus particles or infected cells or through abortive replication. This is similar to what has been observed with SARS-CoV infection, where macrophages can become infected but do not appear to support virus replication (*26*). Abortive replication could result in the production of aberrant replication products that induce a pro-inflammatory response, as has been shown for influenza A virus in the lungs (*27*, *28*).

In the conditions examined here, macrophages appeared to be the major drivers of inflammation in the lungs. This is especially interesting as it has been suggested through the study of healthy human tissue that macrophages interact with ACE2-expressing cells at higher frequencies than other populations (*4*). Both the resident- and monocyte-derived macrophages at 3 dpi were enriched for pro-inflammatory genes, likely caused by having higher titers of virus in the lungs driving the inflammatory state. The MARCO^+^, or tissue-resident, and MARCO^−^, or monocyte-derived, macrophage populations showed distinct patterns of gene expression in the 3- and 10-dpi samples. The MARCO^+^ cells were enriched for interferon-stimulated genes at 3 dpi but were enriched in pathways associated with pro-phagocytic lysosomes only at 10 dpi (*29*). This is unexpected, as MARCO^+^ macrophages have been found to be important in controlling a variety of lung infections (*30*, *31*). Only the MARCO^−^ cells at 3 dpi were enriched in genes associated with migration and lysosomes, suggesting that, at 3 dpi, the nonalveolar macrophages were playing a more prominent role in lung cleanup. Similar alterations have been observed in severe lung conditions such as chronic obstructive pulmonary disease (*32*). Follow-up experiments validating and investigating which macrophage populations are responsible for innate immune host defense and for clearing up the lung environment of cellular debris at different stages after inoculation may help lead to unravel the causes of lung damage and indicate potential targets for therapeutic intervention.

Interferon-stimulated genes represented dominant responses in monocyte and macrophage populations in both the lungs and lymph nodes in the early stages of infection but are reduced by the time infection is cleared. In contrast to the African green monkey model, the role of the interferon response has been proposed as a driver of disease if it is induced with delayed kinetics relative to peak virus replication (*33*, *34*). Single-cell approaches in human peripheral blood mononuclear cells and BALF from patients stratified by COVID-19 severity have revealed that type I, II, and III interferons are sustained at higher amounts in severe patients, whereas interferons declined in moderate patients over time from symptom onset (*6*, *9*, *35*). In the African green monkey model, monocyte recruitment and interferon-stimulated gene responses were temporally controlled along with virus replication. These responses were diminished in the lungs during recovery and were not sustained in the lymph nodes beyond the stage of peak virus replication. Therefore, the African green monkey model reflects effective viral control and reveals transcriptional signatures within tissues associated with protective responses.

This study presents early evidence of changes during a mild SARS-CoV-2 infection in the African green monkey model. One obvious limitation of the animal model is that it does not recapitulate the more severe manifestations of COVID-19 disease. This is an apparent limitation of all current nonhuman primate models of SARS-CoV-2 infection (*10*–*12*, *20*). In addition, this study does not directly address the more specific development of an adaptive immune response due to lack of T or B cell receptor sequencing, especially at 10 dpi. Further studies with this model should be performed not only to increase the sample size being tested but also to include single-cell sequencing of samples of the upper respiratory tract such as nasal brushings and the addition of T and B cell receptor sequencing for an in-depth characterization of the adaptive immune response.

Together, the data reported here provide unique insights into the dynamics of SARS-CoV-2 infection over time in the lungs and associated secondary lymphoid tissue, the identity of the cells hosting replicating virus, and the transcriptional changes in these and other cells during this infectious process. Although the African green monkey model did not result in severe disease upon inoculation with SARS-CoV-2, it did allow for a deeper understanding of virus replication, host response dynamics, and gene signatures associated with a successful resolution of infection. Together, these data can be used to inform development of host-targeted therapeutics for SARS-CoV-2 infection.

## MATERIALS AND METHODS

### Study design

In the search for a nonhuman primate model that recapitulates severe COVID-19, we decided to inoculate African green monkeys with SARS-CoV-2 because they are often used as models for respiratory virus infections and are known to develop acute respiratory distress syndrome when inoculated with Nipah virus (*34*, *35*). To evaluate the pathogenesis of SARS-CoV-2 in African green monkeys, eight adult African green monkeys (four males and four females; body weight, 3.5 to 6 kg) were inoculated via a combination of intranasal (0.5 ml per nostril), intratracheal (4 ml), oral (1 ml), and ocular (0.25 ml per eye) administration of a 4 × 10^5^ TCID_50_/ml (3 × 10^8^ genome copies/ml) virus dilution in sterile Dulbecco’s modified Eagle’s medium (DMEM). Two control animals (one male and one female; body weight, 4.5 to 5.5 kg) were included in the study for comparison. These animals were inoculated via the same routes with the same dose and volume of inoculum. However, the inoculum was γ-irradiated to render the virus noninfectious (*21*). The animals were observed daily (*19*); the same person assessed the animals throughout the study. The predetermined endpoint for this experiment was 3 dpi for the two control animals that were inoculated with irradiated virus and one group of four animals inoculated with infectious SARS-CoV-2, and 10 dpi for the remaining four animals inoculated with infectious SARS-CoV-2. Clinical exams were performed on 0, 1, 3, 5, 7, and 10 dpi on anesthetized animals. On exam days, clinical parameters such as body weight, body temperature, and respiration rate were collected, as well as ventro-dorsal and lateral chest radiographs. Blood and nasal, throat, and rectal swabs were collected during all clinical exams. In addition, on 1, 3, and 5 dpi, animals were intubated and bronchoalveolar lavages were performed using 10- to 20-ml sterile saline. After euthanasia, necropsies were performed. The percentage of gross lung lesions was scored by a board-certified veterinary pathologist, and samples of the following tissues were collected: cervical lymph node, conjunctiva, nasal mucosa, nasal septum, oropharynx, tonsil, trachea, all six lung lobes, mediastinal lymph node, right and left bronchus, heart, liver, spleen, kidney, stomach, duodenum, jejunum, ileum, cecum, colon, and urinary bladder. Histopathological analysis of tissue slides was performed by a board-certified veterinary pathologist blinded to the group assignment of the animals.

All animal experiments were approved by the Institutional Animal Care and Use Committee of Rocky Mountain Laboratories at the National Institutes of Health (NIH) and carried out by certified staff in an Association for Assessment and Accreditation of Laboratory Animal Care International–accredited facility, according to the institution’s guidelines for animal use, following the guidelines and basic principles in the NIH *Guide for the Care and Use of Laboratory Animals*, the Animal Welfare Act, U.S. Department of Agriculture, and the U.S. Public Health Service Policy on Humane Care and Use of Laboratory Animals. African green monkeys were housed in adjacent individual primate cages allowing social interactions, in a climate-controlled room with a fixed light-dark cycle (12-hour light and 12-hour dark). Animals were monitored at least twice daily throughout the experiment. Commercial monkey chow, treats, and fruit were provided twice daily by trained personnel. Water was available ad libitum. Environmental enrichment consisted of a variety of human interaction, manipulanda, commercial toys, videos, and music. The Institutional Biosafety Committee (IBC) approved work with infectious SARS-CoV-2 strains under biosafety level 3 conditions. Sample inactivation was performed according to IBC-approved standard operating procedures for removal of specimens from high containment (*20*, *22*, *36*, *37*).

### Virus and cells

SARS-CoV-2 isolate nCoV-WA1-2020 (MN985325.1) (Vero passage 3) (*38*) was provided by the Centers for Disease Control and Prevention and propagated once in Vero E6 cells in DMEM (Sigma-Aldrich) supplemented with 2% fetal bovine serum (Gibco), 1 mM l-glutamine (Gibco), penicillin (50 U/ml), and streptomycin (50 μg/ml; Gibco) (virus isolation medium). Next-generation sequencing using Illumina MiSeq showed that the used virus stock was 100% identical to the initial deposited GenBank sequence (MN985325.1) with six single-nucleotide polymorphisms detected in 1 to 4% of sequence reads, and no contaminants were detected. Virus was γ-irradiated with a dose of 2 mrad using a JLS Model 484 Co-60 Irradiator to produce a noninfectious inoculum (*22*). Absence of infectious virus after γ-irradiation was confirmed in Vero E6 cells. Vero E6 cells were maintained in DMEM supplemented with 10% fetal calf serum, 1 mM l-glutamine, penicillin (50 U/ml), and streptomycin (50 μg/ml).

### Quantitative PCR

RNA was extracted from swabs and BALF using the QIAamp Viral RNA Kit (Qiagen) according to the manufacturer’s instructions. Tissues (30 mg) were homogenized in RLT buffer, and RNA was extracted using the RNeasy Kit (Qiagen) according to the manufacturer’s instructions. Five microliters of RNA was used in a one-step real-time RT-PCR assay to detect gRNA (forward primer, 5′-ACAGGTACGTTAATAGTTAATAGCGT-3′; reverse primer, 5′-ATATTGCAGCAGTACGCACACA-3′; probe, 5′-FAM-ACACTAGCCATCCTTACTGCGCTTCG-3IABkFQ-3′) and sgRNA (forward primer, 5′-CGATCTCTTGTAGATCTGTTCTC-3′; reverse primer, 5′-ATATTGCAGCAGTACGCACACA-3′; probe, 5′-FAM-ACACTAGCCATCCTTACTGCGCTTCG-ZEN-IBHQ-3′) (*39*, *40*) using the Rotor-Gene Probe Kit (Qiagen) according to the instructions of the manufacturer. In each run, standard dilutions of counted RNA standards were run in parallel to calculate copy numbers in the samples.

### Virus titration and isolation

Virus titrations were performed by endpoint titration in Vero E6 cells. Cells were inoculated with 10-fold serial dilutions of swab and BALF samples. Virus isolation was performed on tissues by homogenizing the tissue in 1 ml of DMEM and inoculating Vero E6 cells in a 24-well plate with 250 μl of cleared homogenate and a 1:10 dilution thereof. One hour after inoculation of cells, the inoculum was removed and replaced with 100 μl (virus titration) or 500 μl (virus isolation) of medium. Six days after inoculation, cytopathic effect (CPE) was scored and the TCID_50_ was calculated.

### Histopathology

Histopathology, IHC, and ISH were performed on African green monkey tissues. After fixation for a minimum of 7 days in 10% neutral-buffered formalin and embedding in paraffin, tissue sections were stained with hematoxylin and eosin. IHC was performed using a custom-made rabbit antiserum against SARS-CoV-2 N at a 1:1000 dilution, using a CD68 clone KP1 mouse monoclonal antibody (Agilent Dako, #M0814) at a 1:100 dilution to identify macrophages, and using Cytokeratin clone AE1/AE3 mouse monoclonal antibody (Agilent Dako, #M3515) at a 1:100 dilution to identify epithelial cells; antibodies were incubated with tissues for 1 hour. Secondary antibodies Discovery OmniMap anti-rabbit horseradish peroxidase (HRP) (Roche Tissue Diagnostics, catalog no. 760-4311 predilute) or Discovery OmniMap anti-mouse HRP (Roche Tissue Diagnostics, catalog no. 760-4310 predilute) were then incubated with the tissues for 15 min. ISH was used for detection of SARS-CoV-2 RNA in selected whole tissue sections of the lungs using the RNAscope VS Universal AP assay (Advanced Cell Diagnostics Inc.) as described previously (*41*) and using probe directed against the SARS-CoV-2 spike (S) gene (catalog no. 848569). Stained slides were analyzed by a board-certified veterinary pathologist.

### scRNA-seq of lung and mediastinal lymph node samples

Lung sections and mediastinal lymph nodes were taken at the time of necropsy and processed. Cell suspensions were generated by manually dicing tissue, enzymatically digesting in RPMI 1640 containing Liberase (0.1 mg/ml; Sigma-Aldrich, 5401127001) and deoxyribonuclease I (0.02 mg/ml; Sigma-Aldrich, 11284932001) at 37°C, and then passing through a 100-μm filter (Becton Dickinson). Suspensions were subjected to ACK lysis and final washes in phosphate-buffered saline containing 0.1% MACS bovine serum albumin (Miltenyi, 130-091-386). A total of 10,000 cells were prepared for 10X Genomics gel bead emulsions. The 10X Genomics version 3.0 chemistry was used. cDNA for the individual cells was generated, and libraries were prepped according to the manufacturer’s protocol. After final libraries were generated, samples were inactivated for any potentially remaining virus using 500 μl of AVL buffer (Qiagen) with 500 μl of ethanol with a sample volume of 140 μl. After a minimum of 10-min incubation, samples were removed from the high-containment laboratory following standard protocols and the libraries were extracted from the AVL using the Qiagen AllPrep DNA spin columns (catalog no. 80204). Samples were then quantified and sequenced. Samples were sequenced on the NextSeq550 using the 10X suggested cycling.

### Processing of scRNA-seq data

Data were processed through the cellRanger pipeline to perform demultiplexing and generate count tables. Alignment was done against the African green monkey Ensembl genome (ChlSab1.1) with the SARS-CoV-2 genome (NC_045512.2) included to be able to parse out reads associated with the viral genome. Samples were then read into R (V3.6.2) using Seurat (V3.1.5) (*16*). Because samples were collected across two different days (day 3 and day 10 after inoculation), we wanted to account for potential batch effects in the global dataset. For this reason, we then integrated the samples using the IntegrateData function. Cells were filtered that contained abnormally high mitochondrial genes (greater than 3 SDs above the median), and cells that were likely doublets were relabeled [ratio of unique features to unique mapped identifier (UMI) per cell < 0.15]. Also, cells containing less than or greater than 3 SDs of UMI compared to the population total were removed to filter for noise. Last, the principal component analysis (PCA) and UMAP projections were calculated for the samples, and clusters of cells were identified. Lung and the lymph node samples were analyzed separately. Gene set enrichment analysis was performed using fgsea (*42*) and the MSig DB (*43*) c2cp gene sets.

### Cell type identification using scRNA-seq data

To determine the identity of either clusters of cells or individual cells, we developed an unbiased method that uses a transcriptional profile of cells instead of a few known marker genes. For the reference data, we used an annotated single-cell sequencing dataset from (*14*). For each of the cell type present in the dataset of lung or spleen tissue, we calculated the differential gene expression using the FindMarkers function in Seurat. To find genes strongly associated with each individual cell type, we filtered the data to contain only those genes with an average logFC (log fold change) greater than 1 and where the difference in the percentage of cells in the cell type of interest expressing the gene compared to the percentage of cells in all other cell types expressing the gene was greater than 0.5. We used this gene set in either the lung or spleen of the human samples (*14*) to develop the marker gene set and calculated the average expression of the marker gene set in each cell type. This generated a matrix of the marker genes to cell types. Then, a correlation of the marker genes in the annotated data was compared to the individual cell or cluster in the African green monkey dataset. This generated a score for the unknown cell or cluster to a known annotation. Using this method, we found that most clusters contained predominately just one cell type. This is a similar method that was developed for the mouse cell atlas (*44*). The results were validated by looking at the expression of the marker genes across the different cell annotations. Our ratios of cell populations was similar to other studies that have performed similar tissue processing steps to collect lung cells from suspension without any enrichment for a given cell type (*14*). When determining the identity on an individual cell level, an additional step was added to help correct falsely identified cells. Using the *k*-nearest neighbors (knn) graph generated in the FindNeighbors function in Seurat, for each cell, its closest neighbors were determined. Once the nearest neighbors were determined, the identities of these neighbors were pulled out. If >70% of the nearest neighbors had one specific identity, the cell identity was reassigned as such. This was run multiple times until a stable number of unidentified cells was found (determined by small changes in unknown cell identities). For those that were not able to be identified, the identity from the original transcriptional profile was used. The cell proportions were found to be fairly consistent between individual animals, suggesting that there were not additional strong batch effects.

Last, to identify clusters that were specifically cells undergoing rapid cell division, we used the CellCycleScore function in Seurat to identify which cell cycle each cell was likely in. We then determined that clusters where greater than 95% of the cells were in G_2_-M or S phase were dividing clusters and were labeled as such.

### SARS-CoV-2 read enrichment

To analyze the enrichment of reads across the SARS-CoV-2 genome, we used Integrative Genome Viewer (*45*) to find read pileups. Cells were labeled as positive for viral RNA if they contained any counts to the viral genome.

### Clustering biases in scRNA-seq data

To determine whether there were clustering biases between two cell types, a new method was developed. Across any cluster of cells, the dataset was subset and renormalized internally to that cluster. Then, the PCs containing up to 99% of the variance explained were calculated. Along each PC, the location of the cells was pulled and grouped on the basis of the two conditions that were being compared. The median location of each of the cell populations along the PC was calculated, and the distance was measured. This was carried out for all PCs and all clusters. To identify outliers with the strongest clustering bias, points outside the mean and 2 SDs across all the PCs and cell types were noted.

### Statistical analysis

Statistical tests comparing cell numbers were carried out in GraphPad Prism version 8 using a one-way analysis of variance (ANOVA). All comparisons between the three groups are reporting adjusted *P* value for multiple testing. A *P* value of 0.05 was considered significant. Statistical tests for gene expression in single-cell data were carried out in Seurat V3.6. Graphs were generated either in ggplot2 (*46*) or in GraphPad Prism version 8.

## SUPPLEMENTARY MATERIALS

stm.sciencemag.org/cgi/content/full/scitranslmed.abe8146/DC1 Fig. S1. Clinical scores, gross lung lesions, and viral loads in respiratory and GI tract of African green monkeys inoculated with SARS-CoV-2. Fig. S2. Histological changes in intestinal tract and lymph nodes of African green monkeys infected with SARS-CoV-2. Fig. S3. Percentage of cell types in the lung determined by scRNA-seq. Fig. S4. Coverage of reads from the pseudo-bulk data across the SARS-CoV-2 genome. Fig. S5. Clustering bias across cell populations in the lungs. Fig. S6. Gene set enrichment analysis of macrophages across the PCs. Fig. S7. Comparison of different cell populations in the mediastinal lymph node. Fig. S8. Analysis of T cell populations in lung and mediastinal lymph node. Table S1. Virus isolation from tissues of African green monkeys inoculated with SARS-CoV-2 and euthanized at 3 and 10 dpi. Data file S1. Raw data. Data file S2. Gene correlations along PCs of the macrophages and interferon stimulated gene (ISG) gene set.

## Appendix

View/request a protocol for this paper from *Bio-protocol*.
